# High-Throughput Metabolomics Discovers Metabolic Biomarkers and Pathways to Evaluating the Efficacy and Exploring Potential Mechanisms of Osthole Against Osteoporosis Based on UPLC/Q-TOF-MS Coupled With Multivariate Data Analysis

**DOI:** 10.3389/fphar.2020.00741

**Published:** 2020-05-20

**Authors:** Zhenxing Si, Shifeng Zhou, Zilong Shen, Feiyu Luan

**Affiliations:** ^1^Emergency Surgery, First Affiliated Hospital of Harbin Medical University, Harbin, China; ^2^Orthopedic Department, Second Affiliated Hospital of Harbin Medical University, Harbin, China

**Keywords:** metabolomics, osteoporosis, natural product, metabolic profiles, biomarker, pathway

## Abstract

Postmenopausal osteoporosis (PMOP) is the most common metabolic bone illness among the elderly especially in postmenopausal women resulting from a reduction in bone mineral density, but there is no effective drug at present. The study was aimed at evaluating efficacy of osthole against osteoporosis using high-throughput metabolomics method. The blood samples for illustrating the pathological mechanism of PMOP and exploring the efficacy of osthole treatment (ST) were collected to perform metabolites and metabolic profiles and pathways analysis using ultra-performance liquid chromatography coupled with quadrupole time-of-flight mass spectrometry (UPLC-Q-TOF/MS) and pattern recognition methods. In addition, backbone weight, the bone density, and some vital biochemical indexes were also detected. A total of 28 metabolites were identified as biomarkers for ovariectomized-osteoporosis model, and ST could significantly regulate 19 of them including lysine, linoleic acid, 3-hydroxybutyric acid, prostaglandin F2a, taurocholic acid, LysoPC(15:0), l-carnitine, glucose, arginine, citric acid, corticosterone, ornithine, tryptophan, arachidonic acid, Cer(d18:0/18:0), glutamine, uric acid, 8-HETE, estriol, which mainly related with 13 metabolic pathways, such as linoleic acid metabolism, starch, and sucrose metabolism, arachidonic acid metabolism, alanine, aspartate and glutamate metabolism, arginine and proline metabolism, citrate cycle (TCA cycle), and arginine biosynthesis. The ovariectomized model (OVX) rats display a significant decrease bone density, TGF-β1, NO, and NOS level, and a significant increase bone weight, IL-6, TNF-α, and Ca ^2+^ level. These parameters in the ST rats were evidently improved as compared to the OVX group. ST effectively mitigated ovariectomy-induced osteoporosis in rats by affecting endogenous metabolite-related metabolic mechanism and showed the natural alternative with potential for the treatment of PMOP.

## Introduction

Osteoporosis is a progressive bone illness characterized by reduced bone mass and density, microarchitectural deterioration of tissue and proteins expression alteration in bones (Sun et al., 2013a; Aujla and Majithia, 2016; Qiu et al., 2019). Women with all ages resulting from physiological structure of bone as well as gradually reducing levels of estrogen and progesterone, which is related with higher rate of bone resorption in osteoclasts are approximately five times possibility to suffer from osteoporosis than men (Srivastava and Deal, 2002; Adler, 2014). It was reported that about 200 million women aged between 45 and 55 years are affected by postmenopausal osteoporosis (Hromadnikova et al., 2020). Bone mineral density (BMD) as an essential indicator in clinical practice to appraise osteoporosis is not sensitive to the patients with severe ostealgia and humpback leading to the morbidity rate continues to increase including young adults (Ha et al., 2020; Guo et al., 2020). Hormone replacement therapy is commonly applied to protect against osteoporosis in addition to conventional medicines. Currently, the combination estrogen combined with calcium therapy is more and more popular with people (Tarakida et al., 2008; Gambacciani and Levancini, 2014). However, the serious side effects, such as thrombosis, hypertension, atherosclerosis, organ necrosis, and even cancer, are unavoidable (Tárraga López et al., 2011; Maichuk et al., 2014; Hamood et al., 2019; Lou et al., 2019). Therefore, there is an urgent need to discover and develop emerging methods containing natural products with minimal side effects and strong activity for prevention and treatment of osteoporosis in early stage.

Traditional Chinese herb has exhibited treatment and prevention effect on diseases (Zhang et al., 2020). Cnidium is a dried and mature fruit of the umbelliferous plant *Cnidium monnieri* (L.) Cuss. The main active ingredient of Cnidium are the coumarins, of which the content of osthole is the highest. Cnidium has great potential in the treatment of OP (Xie et al., 1994; Liao et al., 1997). The traditional Chinese compound containing cnidium could facilitate the proliferation and differentiation of osteoblastic cells UMR106 and enhance the bone density of ovariectomized rats. Total coumarins could inhibit the differentiation of osteoclast precursor cells into osteoclasts for restraining the bone resorption of mature osteoclasts. At the molecular level, osthole can regulate the JNK pathway by inhibiting the expression of related proteins level to stimulate osteoclast apoptosis and restrain bone resorption. Other reports have found that osthole possess ability to prohibit osteoclast conformation and partly deteriorate bone loss in nephrectomized mice by significantly increasing the expression of osteogenic differentiation-related factors and promoting Osteoprotegerin (OPG) secretion and calcium salt deposition (Li et al., 2002; Ming et al., 2011; Wang et al., 2018; Zheng et al., 2019). It suggested that osthole has medicinal value in promoting osteoblast bone formation.

Metabolomics as an instantly emerging “omics” device points to qualitative and quantitative analysis of endogenic small metabolites in cells, tissues, organs or biological fluids, and help understand the metabolomic profiling changes indicating the details of the underlying pathways in response to endogenous and exogenous impetus, such as food, drug, and pathogen (Sakaguchi et al., 2019; Xie et al., 2019; Xiong et al., 2019). Recently, modern high-throughput technology including nuclear magnetic resonance spectroscopy (NMR), gas chromatography mass spectrometry (GC-MS), liquid chromatography mass spectrometry (LC-MS), or capillary electro-phoresis mass spectrometry (CE-MS) coupled with multivariate analysis was successfully performed multiparametric metabolic responses to pathophysiological and environmental perturbations in metabolomics research (Sun et al., 2013b; Zhang et al., 2017; Sun et al., 2019; Zhao et al., 2020). It has been widely used in early diagnosis, disease staging, and treatment evaluation of osteoporosis (Lv et al., 2016; Zhao et al., 2018; Xia et al., 2019; Zhang et al., 2019; Ye et al., 2020). Animal model of OP can be set up by traditional castration surgery and modern study (Tella and Gallagher, 2014; Drake et al., 2015). In this study, a serum metabolomics method based on UPLC/Q-TOF-MS coupled with multivariate data method and pathway analysis was applied to depict the changes of biomarkers, metabolic profiles, and pathways as potential targets for evaluating natural product osthole pharmacodynamic effect in ovariectomized-induced osteoporosis rats. The result was found that ST effectively mitigated ovariectomy-induced osteoporosis in rats by affecting 19 endogenous metabolites related with 13 metabolic pathways, such as linoleic acid metabolism, starch and sucrose metabolism, and arachidonic acid metabolism, which showed the natural alternative with potential for the treatment of PMOP.

## Experimental Section

### Materials

MS grade acetonitrile and methanol were purchased from Merck KGaA (Darmstadt, Germany), and formic acid was gained from Fisher Scientific (Pittsburgh, PA, USA). Ultra-high purity water (18.2 MΩ) was purchased from a Millipore SAS-67120 (Molsheim, Cedex, France). Leucine enkephalin was obtained from Sigma-Aldrich (St. Louis, MO, USA). Alendronate and penicillin were provided by DELPHARM (Lille, France). Osthole substance (batch No. 120710-201809) with 99.2% purity was provided by CSPC Zhongnuo Pharmaceutical (Shijiazhuang City, China) and its HPLC characteristic chromatography was shown in **Supplementary Material, Figure S1**. Chloral hydrate, physiologic saline solution, and 95% alcohol solution was bought from Tong Ren Tang Chinese Medicine Co., Ltd. (Beijing, China). The kits for interleukin-6 (IL-6), tumor cell necrosis factor-alpha (TNF-α), transformation growth factor-β1 (TGF-β1), nitric oxide (NO), and nitric oxide synthase (NOS) detection were offered from DELPHARM (Lille, France). The kits for serum calcium (Ca^2+^), magnesium (Mg^2+^), and phosphorus (P^2−^) detection were purchased from GIBCO (Grand Island, NY). The kits of serum alkaline phosphatase (AKP) and osteocalcin (OC) were gained from Ludwig Biotecnologia Ltda (Alvorada, RS, Brazil). Other agents not mentioned were of analytical grade in this experiment.

### Animals and Modeling Method

The Animal Ethics Committee of Harbin Medical University ratify the experimental protocol and animals feeding method in this experiment. A total of 40 female SD rats (5-week-old, weighing 260 ± 20 g) were gained from DBL, Inc. (Eumseong, Korea). Animals were accommodated in a temperature-controlled room environment (24 ± 2°C) with constant humidity (45% ± 5%) and a 12/12-h light/dark cycle, which were optionally fed standard research food and water for a seven days adaptation period. One week later, rats were indiscriminately split into four groups consisting of ten rats every group, as follows: sham-operated (SHA), ovariectomized model (OVX), ovariectomized rats with osthole treatment (OVX+ST), ovariectomized rats with alendronate treatment (OVX+ALE). Except for the SHA group, the unsettled 30 rats were carried out 12-h fasting until surgery and then general hocused with 10% chloral hydrate (3 ml/kg). Bilateral ovariectomy was performed in female rats through an incision in the abdomen, which about a 2-cm-long, 2- to 2.5-cm-deep incision is cut longitudinally from the white line of the abdomen to separate abdominal cavity and muscles. The ovaries are exposed, and then, the uterine horn is put back to the abdominal cavity. The oviduct was ligated by a silk thread, and the other side is operated in the same way. The muscle and skin layers were sutured with medical biological thread, and then disinfected with iodophor (Kim et al., 2019; Maiz et al., 2019; Zongmin et al., 2020). Rats in the SHA group were performed a sham surger under the same protocol. Penicillin sodium in a dose of 50,000 units was injected into the abdominal cavity for anti-inflammatory and wound healing.

### Treatment

Osthole was disintegrated with 0.9% sodium chloride to prepare a concentration of 100 mg/ml solution. At 4 weeks before modeling and eight consecutive weeks after modeling, the OVX+ST group was cured with 400 mg/kg osthole every day, and the OVX+ALE group was treated with 1 mg/kg alendronate every day. The SHA group and OVX group were given equal amounts of 0.9% sodium chloride orally. The drug dose was calculated and updated once a week on the basis of the body weight.

### Specimen Collection and Routine Index Detection

After 3 months of drug intervention, all the rats were softly hocused with chloral hydrate and then 5-ml blood was fetched by heart puncture. The blood sample was stored in serum tubes right away and centrifuged at 3,500 rpm for 15 min at 4°C. The extracted serum was applied to metabolomics analysis and routine index detection (Sareen and Jack, 2013; Fabien et al., 2015; Ran et al., 2019; Spring et al., 2020). The serum contents IL-6, TNF-α, TGF-β1, NO, NOS, Ca^2+^, Mg^2+^, P^2−^, AKP, OC were detected by radioimmunoassay according to the instruction in the kits. The femur of the rat was taken, and the dry weight was measured. The soft tissue and muscle was removed from the femur and fixed with 95% alcohol. Dual-energy X-ray absorptiometry (DSC-3000, Aloka, Tokyo, Japan) was employed to detect the bone density of the experimental rats after oral treatment.

### Instrumental Analysis

#### Sample Preparation

Before analysis, the serum samples were thawed at 4°C and extracted by adding 900 µl of ice-cold methanol to removal of protein. The mixture was integrated 30 s by a mixer mill (Retsch GmbH & Co, Haan, Germany), then they were centrifuged at 15,800*g* for 15 min at 4°C. The gained upper liquid were delivered into a new centrifuge tube and vaporized to dryness under nitrogen at 24 ± 2°C. After reconstituting 150 μl of water, the samples were again detached at 15,800*g* for 15 min at 4°C. They were passed through a 0.22-μm PTFE filter to UPLC/Q-TOF-MS analysis. Quality control (QC) sample was applied to ratify and optimize the chromatographic and MS condition, which was prepared by mixing an equal aliquot 8 μl from all groups.

#### Analysis of UPLC/Q-TOF-MS

UPLC ACQUITY™ System (Waters, Milford, MA, USA) combined with a Waters Micromass Q-TOF Premier was employed to resolve serum sample, which was first separated ACQUITY UPLCTM HSS T3 (100 mm × 2.1 mm id, 1.8 µm) followed by absorbance measurements using an ACQUITY™ UPLC Tunable UV (TUV) Detector (Waters). Mobile phase was comprised of phase A and phase B, which were, respectively, acetonitrile containing 0.05% formic acid and water containing 0.05% formic acid. The gradient program was set as follows: 0–1 min, isocratic 2% A; 1–4 min, linear gradient from 2% to 40% A; 4–5 min, isocratic 40% A; 5–7 min, 40% to 70% A; 7–9 min; 70% to 90% A; 9–10 min; 90% to 100% A; isocratic 100% A for 2 min and afterward back to 2% A in 3 min. The flow rate was kept at 0.3 ml/min, and the injection volume of samples was 4 µL. Electrospray ionization source (ESI) was performed for a 100 to 1,000 m/z full-scan in positive and negative ion mode. The critical parameters of Q-TOF-MS were set as follows: capillary voltage was 5.0 kV in positive ion mode and 4.0 kV in negative ion mode; source temperature of 300°C; desolvation temperature of 450°C; the deconvolution voltage (DP) is set 140 V, and the collision energy (CE) is set to 40 eV; the sample cone voltage of 35 V both in both ion mode; Nitrogen gas is used as the atomizing gas and the auxiliary gas, which the flow of 900 L/h, 60 psi and 55 psi were, respectively, in positive and negative ion modes. In the process of MS analysis, 300 pg/ml leucine-enkephalin calibration solution that was [M-H]^−^ = 554.2615 and [M + H]^−^ = 556.2771 was constantly infused into the MS at 4 μl/min flow-rate to maintain the preciseness and reproducibility of the TOF-MS.

#### Data Processing

In light of the established 10-min gradient program, the serum of rats was analyzed by UPLC/Q-TOF-MS technology in both ion mode. The preprocessed data were converted to NetCDF format (*.cdf) using DataBridge (Waters) software, and then the main information, such as the mass, retention time, and intensity of the peaks in each chromatogram, were collected by MarkerLynx XS application manager software (version 4.1, Waters, Manchester UK). After peak alignment and normalization process, the obtained files including peak code, m/z value, and corresponding normalized intensities was transferred to Excel sheets for multivariate pattern recognition analysis using SIMCA-P software (version 13.0, Umetrics, Umeå, Sweden). Unsupervised principal component analysis (PCA) was carried out on all ions to gain a general view and determine the ions with singularity value. Orthogonal partial least squares-discriminant analysis (OPLS-DA) as a supervised method is utilized to discover a model that was calculated the classes of observations of the Y axis (or vertical line) based on their X variables (or horizontal line). The cumulative goodness of the OPLS-DA models was estimated by permutation tests, which the parameters R2 (cum) and Q2 (cum), respectively, represent the fitness and predictive ability. Pareto scaling was utilized for PCA and OPLS score plot analysis during data processing. Statistically significant ions that meet the variable importance projection (VIP) value greater than 1.2 and a *P* value less than 0.05 in Statistica 7 (StatSoft, Tulsa, OK, USA) were deemed as potential metabolites. The metabolites were identified by the combination of retention times, standard compounds comparison, MS/MS data, and online database, such as MycompoundID (http://www.mycompoundid.org), MassBank (http://www.massbank.jp), ChemSpider database (www.chemspider.com), and METLIN (http://metlin.scripps.edu). MetPA in MetaboAnalyst 4.0 (https://www.metaboanalyst.ca/) combines with various advanced path analysis programs to resolve the topological trait of metabolite metabolic pathways for highlight differential biomarker role in biological system.

### Statistical Analysis

Statistical analysis of variance (ANOVA) for the differences evaluation was applied to SPSS software, version 12.0 (SPSS, Inc., Chicago, IL, USA) and GraphPad Prism 6.0 (La Jolla, CA, USA). Data are presented in mean ± standard deviation way. A value of *P* < 0.05 was considered to indicate a statistically difference, and a value of *P* < 0.01 was considered to indicate a remarkably statistically difference.

## Results

### Dry Weight and Bone Density of Femur Analysis

**Figure 1:**
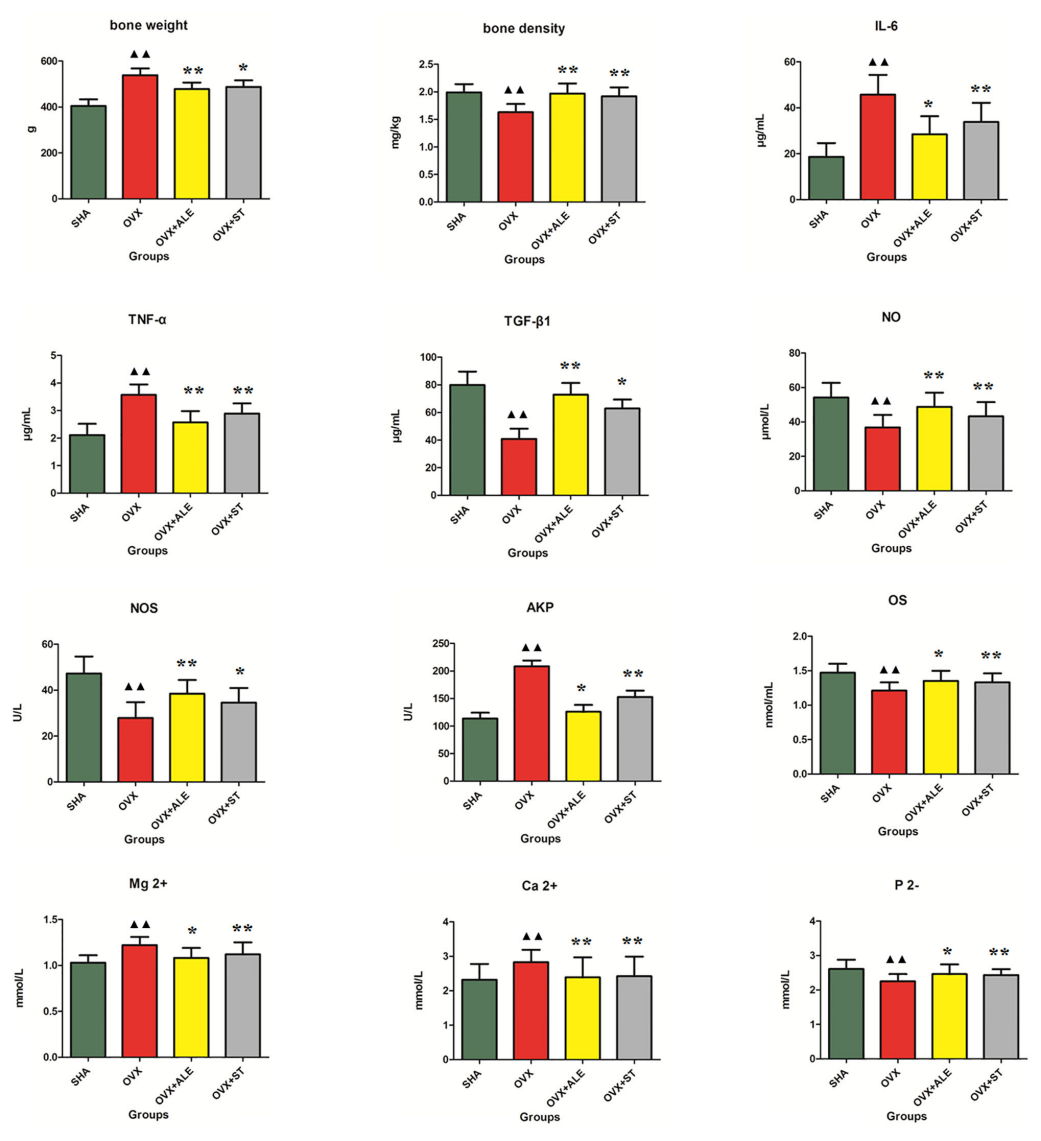
Effects of ST on bone weight, bone density, TGF-β1, NO, NOS, OS, P 2-, IL-6, TNF-α, AKP, Ca^2+^, Mg^2+^ level of ovariectomized osteoporosis rat model. Compared with OVX model group, *P < 0.05, **P < 0.01. Compared with control group, ^▲▲^*P* < 0.01 was considered to indicate a remarkably statistically difference.

Osteoporosis is a skeletal abnormality characterized by the reduction of bone strength leading to the increasing risk of fractures. Bone strength reflects the two role of bone density and bone mass and is of great significance for assessing the degree of osteoporosis. Some studies reported that dry weight of bone showed an increasing trend, indicating that osteoporosis symptoms in rats were obvious. In **Figure 1**, the femoral dry weight and bone density of the model group rats were higher than those of the SHA group, which was preliminarily judged that osteoporosis rat model establishment was successful. After 3 months of ST treatment, the femoral dry weight and bone mineral density of the rats in the OVX+ST group were increased, indicating that ST can improve osteoporosis.

### Serum Immunoregulatory Factor and TGF-β1 Analysis

Serum immunomodulatory factors IL-6 and TNF-α can aggravate osteoporosis. TGF-β1 can promote bone cells division, up-regulation, and proliferation capacity. From **Figure 1**, the IL-6 and TNF-α level in model group rats were higher than those of the SHA group, and the TGF-β1 content was lower than those of the SHA group. After 3 months of ST treatment, the IL-6 and TNF-α levels of the rats in the OVX+ST group were reduced, and the TGF-β1 content was higher than model rats, indicating that ST can improve osteoporosis, which the effect is slightly lower than ALE.

### Serum Ca^2+^, Mg^2+^, and P^2-^ Analysis

When osteoporosis occurs in the body, bone resorption can lead to an increase in positive divalent calcium and magnesium ions in the serum and a decrease in negative divalent phosphorus ions. In **Figure 1**, serum Ca2+ and Mg2+ content in model rats were higher than those of the SHA group, and the P^2-^ level was lower than those of the SHA group. After 3 months of ST treatment, serum Ca2+ and Mg2+ content of the rats in the OVX+ST group were reduced and the P^2-^ level was higher than model rats, indicating that ST can improve osteoporosis, which the effect is moderately lower than ALE.

### Serum NO, NOS, AKP, and OC Analysis

NO is an important signaling and effector molecule in the body, and NOS is the rate-limiting enzyme for NO synthesis. In bone tissue, NO produced by bone cells can affect bone reconstruction through autocrine or paracrine way. Under pathological conditions, abnormal secretion of NO can cause bone formation and resorption imbalance, which is closely related to the pathogenesis of osteoporosis. The higher the content of nitric oxide and nitric oxide synthase in serum, the better the effect of osteoporosis treatment. OS can directly reflect bone development and metabolism, both of which show positive growth relationship. AKP as an enzyme was widely distributed in the human liver, bones, intestines, kidneys, placenta, and other tissues, and excreted from the liver to the bile. Alkaline phosphatase (AKP) was positively correlated with osteoclasts. In modern clinical practice, AKP and OS in serum are often used as the basis indicators for diagnosis. Therefore, the decreasing content of OS and the increasing content of AKP in serum when osteoporosis occurs. In **Figure 1**, serum AKP content in model rats were higher than those of the SHA group, and the NO, NOS, and OS levels were lower than those of the SHA group, indicating osteoporosis rat model establishment was successful. After 3 months of ST treatment, serum AKP content of the rats in the OVX+ST group were reduced, and the NO, NOS, and OS levels were higher than the model rats, indicating that ST can improve osteoporosis.

### Metabolic Profiling Analysis

The gained QC sample was analyzed three times during the analytical run to ensure system equilibration and then was repeated once every six samples for assuring the notably differences ascended from intrinsic discrepancy between groups rather than instrumental movement. In positive and negative ion modes, more than 90% of detected variables had relative standard deviation (RSD) values less than 30% suggesting that the analytical method possess outstanding repeatability and stability for metabolomics study. Under the optimal elution program, the serum metabolites of SHA, OVX, OVX+ST, OVX+ALE groups were profiled.

**Figure 2:**
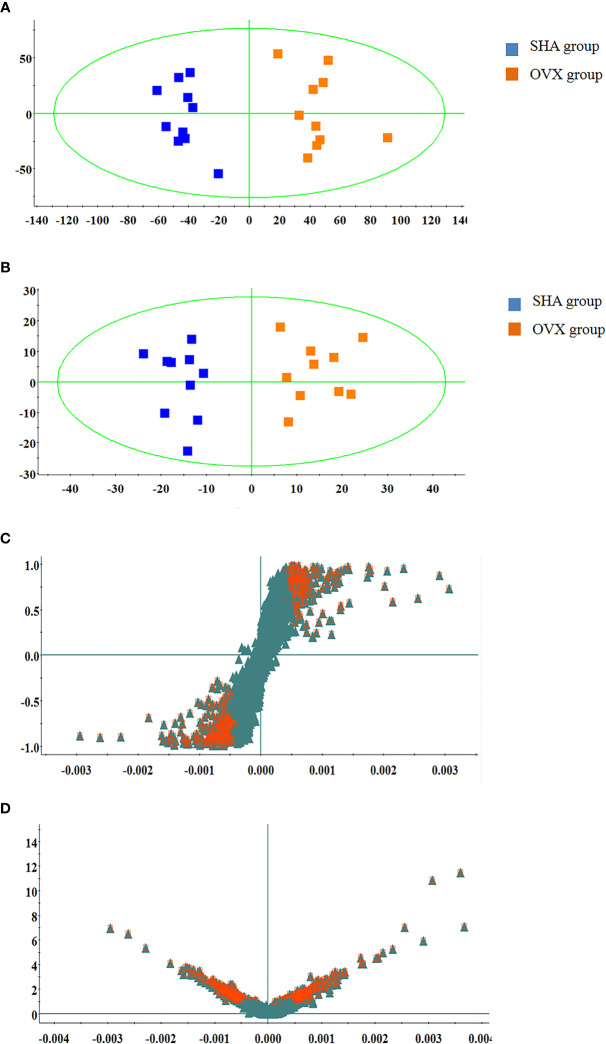
Multivariate statistical analysis of UPLC-MS data in positive ion mode. **(A)** PCA score plot; **(B)** OPLS-DA score plot; **(C)** S-plot of OPLS-DA model; **(D)** VIP-plot of OPLS-DA model.

**Figure 3:**
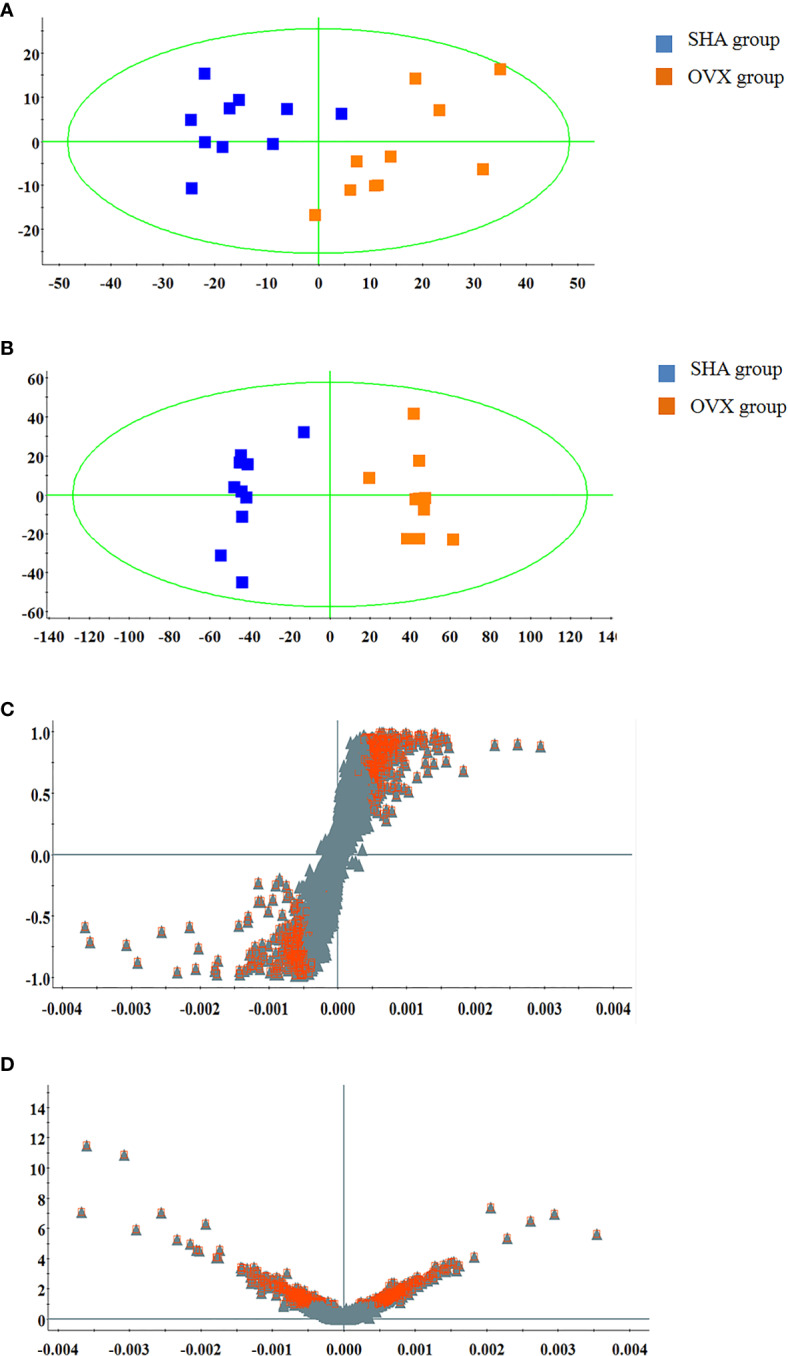
Multivariate statistical analysis of UPLC-MS data in negative ion mode. **(A)** PCA score plot; **(B)** OPLS-DA score plot; **(C)** S-plot of OPLS-DA model; **(D)** VIP-plot of OPLS-DA model.

The serum UPLC/Q-TOF-MS metabolic profile data after normalization processing were input into the SIMCA-P. All ions were resolved in PCA score plot to obtain the change between SHA and OVX group, which each point corresponds an individual blood sample. As shown in **Figures 2A** and **3A**, the clustering trend displayed that the SHA group was separated evidently from OVX group in a direction away demonstrating that the serum metabolic state of animals after ovariectomized operation changed notably. In order to select differentially expressed metabolites, the obtained metabolic profile of serum sample was resolved by a more sophisticated OPLS-DA, which the clustering of OVX model rats was clearly separated from the SHA rats in **Figures 2B** and **3B**. From the S-plot diagram obtained from OPLS-DA analysis showed in positive (**Figure 2C**) and negative ion mode (**Figure 3C**), the ions farther away from the origin point, the greater the contribution rate in animals model establishment. Further select ions in VIP scatter plot that highlight the variation and correlation contribution by VIP value were illustrated in the positive mode (**Figure 2D**) and negative mode (**Figure 3D**). Potential biomarkers were judged though the combination with the VIP value more than 1.5 and P value of <0.05 in inter-group T-test.

**Figure 4:**
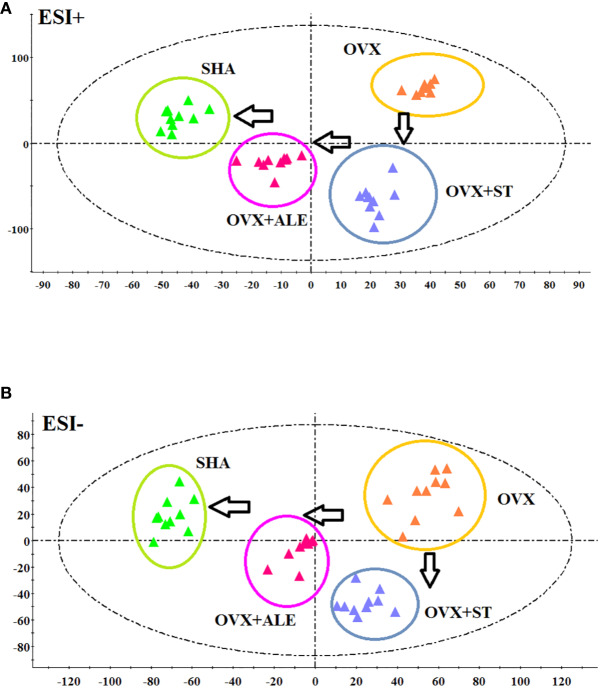
PCA scores plot of metabolic profiling of the ovariectomized osteoporosis rat model in both positive and negative mode. **(A)** In positive ion mode and **(B)** negative ion mode.

**Figure 4** demonstrates the PCA score plot that the tendency of alteration between SHA, OVX, OVX+ST, OVX+ALE groups. It can be seen that the clusters in every group were gathered and detached among groups. Samples from sham-operated rats was greatly separated from OVX model suggesting that serum metabolism profiling of animals after model replication was distinct from that of the SHA rats. Samples from OVX+ST and OVX+ALE groups were situated between the SHA group and the OVX group, which the clustering of OVX+ALE groups is closer to the SHA group. The behavior of the OVX + ST group is similar to that of the OVX + ALE group, and the trend is slightly inferior to that of the OVX + ALE group. It was indicated that ST could postpone the pathological process of ovariectomized osteoporosis and promote metabolic state of model rats to normal levels.

### Metabolite Identification and Analysis

**Figure 5:**
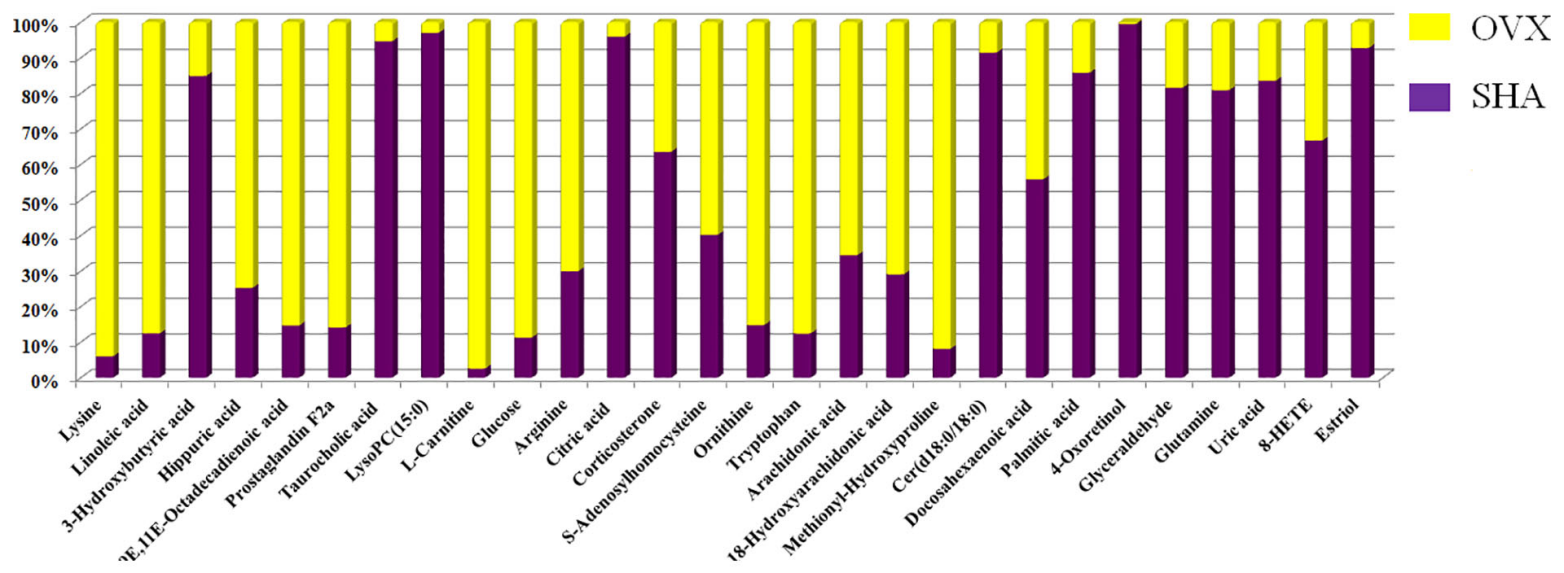
The relative proportion changes of metabolites between SHA and OVX group by histogram calculated using the peak area.

**Figure 6:**
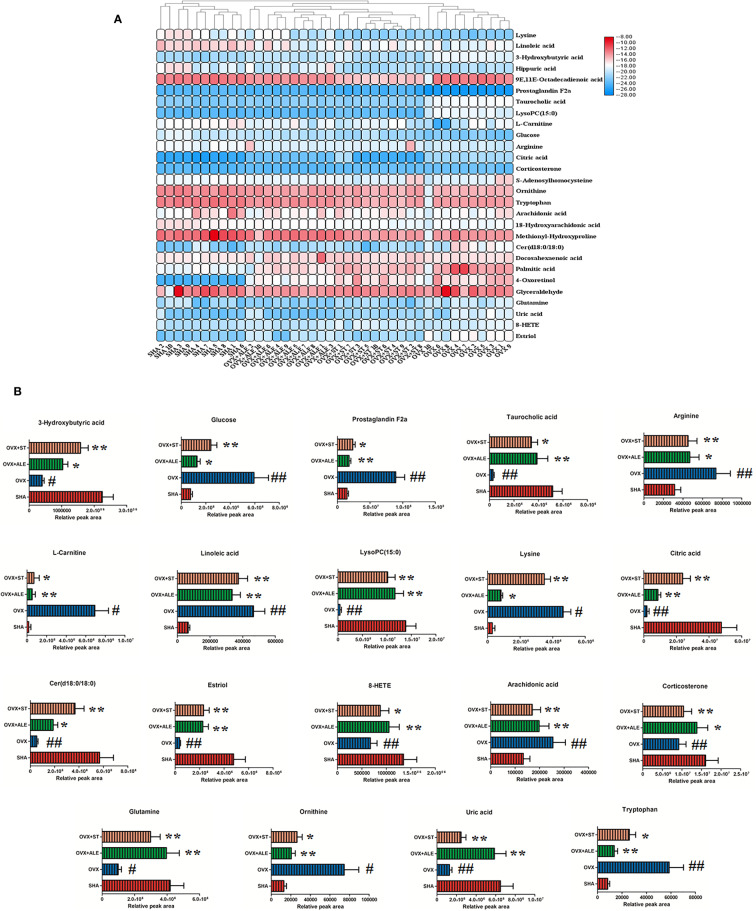
Heatmap with different color changes to highlight relative content alteration of the metabolites from SHA, OVX, OVX+ST, OVX+ALE groups **(A)**. Bar chart presents the relative peak area of above mentioned metabolites **(B)**. Compared with SHA group, ^#^*P* < 0.05, ^##^*P* < 0.01. Compared with OVX model group, *P < 0.05, **P < 0.01.

The chemical compositions and presume molecular formula of differential ions with VIP more than 1.2 and *P* value less than 0.05 were affirmed by MS/MS data information, such as Rt, accurate mass, error (mDa), and available biochemical databases. In total, 28 metabolites, including lysine, linoleic acid, 3-hydroxybutyric acid, hippuric acid, 9E,11E-octadecadienoic acid, prostaglandin F2a, taurocholic acid, LysoPC(15:0), l-carnitine, glucose, arginine, citric acid, corticosterone, S-adenosylhomocysteine, ornithine, tryptophan arachidonic acid, 18-hydroxyarachidonic acid, methionyl-hydroxyproline, Cer(d18:0/18:0), docosahexaenoic acid, palmitic acid, 4-oxoretinol, glyceraldehyde, glutamine, uric acid, 8-HETE, estriol, were identified and summarized in **Supplementary Material, Table S1** for contributing to the SHA and OVS group separations. As shown in **Figure 5**, the relative proportion of metabolites among the two experimental groups are displayed by histogram calculated using the peak area. Compared with sham-operated rats, fourteen metabolites, lysine, linoleic acid, hippuric acid, 9E,11E-octadecadienoic acid, prostaglandin F2a, l-carnitine, glucose, arginine, S-adenosylhomocysteine, ornithine, tryptophan, arachidonic acid, 18-hydroxyarachidonic acid and methionyl-hydroxyproline level were higher in OVS model rats. Meanwhile, other 13 metabolites content were lower in OVS model rats. In order to uncover the visualization changes of potential biomarkers of model rats after ST and ALE positive control administration, a heatmap was built in **Figure 6A** with color differences to highlight the metabolites alteration. It was easy to be seen that ST treatment could affect 19 metabolites, including lysine, linoleic acid, 3-hydroxybutyric acid, prostaglandin F2a, taurocholic acid, LysoPC(15:0), l-carnitine, glucose, arginine, citric acid, corticosterone, ornithine, tryptophan, arachidonic acid, Cer(d18:0/18:0), glutamine, uric acid, 8-HETE and estriol, which the color is regulated back in varying degrees and trend to SHA group. The relative peak area of abovementioned metabolites were shown in **Figure 6B**, among them, six metabolites were statistically different (*P* < 0.05), and 13 metabolites were dramatically statistically different (P< 0.01).

### Metabolic Pathways Analysis

**Figure 7:**
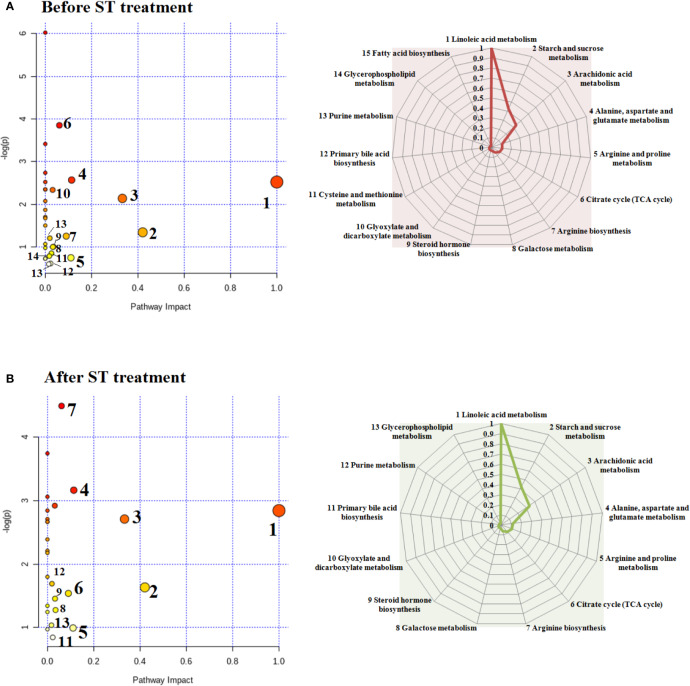
Changes in endogenous metabolic pathways before and after ST treatment and the corresponding influence values in the spider web. Before ST treatment, fifteen metabolic pathways with p value more than zero in animals model **(A)**. After ST treatment, thirteen metabolic pathways with p value more than zero in animals model **(B)**.

The appraised biomarkers of ovariectomized-induced osteoporosis were entered into MetPA application in MetaboAnalyst 4.0 (https://www.metaboanalyst.ca/) to carry out pathway and molecular networks construction with biological background, which have ability to judge some metabolic pathway most relevant to metabolomics study. Before ST treatment, fifteen metabolic pathways with p value more than zero including linoleic acid metabolism, starch and sucrose metabolism, arachidonic acid metabolism, alanine, aspartate and glutamate metabolism, arginine and proline metabolism, citrate cycle (TCA cycle), arginine biosynthesis, galactose metabolism, steroid hormone biosynthesis, glyoxylate and dicarboxylate metabolism, cysteine and methionine metabolism, primary bile acid biosynthesis, purine metabolism, glycerophospholipid metabolism as well as fatty acid biosynthesis were shown in **Figure 7A**. Linoleic acid metabolism with impact values of one were deemed as the critical disturbed pathways in progression of disease. After ST treatment, 13 metabolic pathways including linoleic acid metabolism, starch and sucrose metabolism, arachidonic acid metabolism, alanine, aspartate and glutamate metabolism, arginine and proline metabolism, citrate cycle (TCA cycle), arginine biosynthesis, galactose metabolism, steroid hormone biosynthesis, glyoxylate and dicarboxylate metabolism, primary bile acid biosynthesis, purine metabolism, glycerophospholipid metabolism were shown in **Figure 7B**, which were deemed as the critical metabolic pathways closely associated with ST pharmacodynamic effects.

## Discussion

Metabolomics strategy was used to analyze the endogenous differential small molecule metabolites in ovarian osteoporosis model rats and control rats. At the eighth week after model replication, the clustering of OVX group and the SHA group are separated, which the difference reflects in the level of 28 endogenous metabolites involved in amino acid metabolism, lipid metabolism, carbohydrate metabolism, TCA cycle, purine metabolism, bile acid metabolism, and others implying that the above metabolic pathways may be latent biochemical mechanisms of PMOP. After oral administration of ST, it was initially determined that ST can recall the abnormal levels of 19 metabolites, mainly related to 13 metabolic pathways such as linoleic acid metabolism, starch and sucrose metabolism, arachidonic acid metabolism, alanine, aspartate and glutamate metabolism, arginine and proline metabolism, and citrate cycle (TCA cycle).

Unsaturated fatty acids as an essential fatty acids make up fat for the body. Among food fats, monounsaturated fatty acids include oleic acid, and polyunsaturated fatty acids include linoleic acid, linolenic acid, and arachidonic acid. In the light of the position and function of the double bond, polyunsaturated fatty acids (PUFA) are divided into omega-6 series and omega-3 series, which linoleic acid and arachidonic acid belong to the omega-6 series, meanwhile linoleic acid can be converted into arachidonic acid in the body. Studies have found that PUFA affects bone metabolism through various mechanisms (Ilich et al., 2014). Arachidic acids are metabolites of PUFA and are signaling molecules for immune and inflammatory responses *in vivo*. The main metabolites of n-6 PUFAs are pro-inflammatory arachidic acids, including prostaglandin E2 (PGE2), leukotriene B4 (Leukotriene B4, LBT), and thromboxane A2 (Thromboxane A2, TXA2), which these products are produced by lipoxygenase-5 (5-lipoxygenase, 5-LOX) and cyclooxygenase- (cyclooxgenase-2, COX-2) (Kelly et al., 2013). During metabolism, n-6 PUFAs compete with n-3 PUFAs for capturing saturated enzymes that produce anti-inflammatory responses (Liu et al., 2006). The higher the level of n-6 PUFAs in a modern diet, the more pro-inflammatory response of arachidonic acid equilibrium (Bagga et al., 2003). The high level of n-6 PUFA promotes the formation of osteoporosis. On the one hand, n-6 PUFA promoted the differentiation of MSCs into adipocytes, inhibited the differentiation of MSCs into osteoblasts, and reduced bone formation (You et al., 2014). On the other hand, n-6 PUFA stimulated the production of inflammatory cytokines and activated osteoclast cells to increase bone resorption. n-6 PUFA up-regulates the expression of COX-2, which increases the production of PGE 2 and inhibits the formation of bone matrix (Liu et al., 2012). This study found that serum linoleic acid, prostaglandin F2a, and arachidonic acid were abnormally elevated in ovariectomized osteoporotic rats. It is speculated that the biosynthesis of unsaturated fatty acids is destroyed leading to osteoporosis. In the formation of postmenopausal osteoporosis, amino acid metabolism is disturbed. Glutamine may regulate bone metabolism through osteoclasts and can be converted into glutamate by each other. Glutamate may cause bone resorption through the expression of glutamate receptors on bone cells, which could explain the association between elevated glutamine and low content of BMD (Huang et al., 2016). It was found that arginine, phenylalanine, tryptophan, and valine levels were elevated, and creatine levels were reduced in serum samples. The results may be related to increase NO production, reduced stimulation of hormones and insulin-like growth factor-1, and excessive muscle breakdown, which may be responsible for the development of osteoporosis (Hall and Greendale, 1998; Fini et al., 2001). Lysine is one of the essential amino acids in the human body, which can promote human development, enhance immune function, and improve the function of central nervous tissue. L-lysine is an essential factor for bone collagen synthesis, and its deficiency will lead to reduced bone collagen synthesis. It can not only affect bone collagen metabolism, but also change physical properties of bone collagen fibers (Cheng et al., 1992). Fini M found that the synthesis of N-type collagen was increased by adding arginine and lysine in cultured osteoporotic rat osteoblast in comparison with the control group (Tsai et al., 2015). As an amino acid that guides the urea cycle, ornithine increases insulin and hormone levels. The body needs it to build and maintain muscle during physical training, and it helps reduce muscle loss during aging. With age, the body's efficiency in protein synthesis will decrease, and muscle tissue regeneration capacity will decrease. By increasing the growth hormone levels, ornithine assists in accelerating muscle tissue production and delaying the effects of aging (Lee et al., 2016; Dirckx et al., 2018). This study found that the lysine, arginine, ornithine, tryptophan content in the model group rats increased abnormally, meanwhile the glutamine content decreased, indicating that amino acid metabolism disorders occur in osteoporotic rats.

Osteoporosis is closely related to diabetes, and imbalance of bone reconstruction in postmenopausal osteoporosis can also cause glucose intolerance and insulin resistance. Dirckx et al. found that VHL gene knockout mice significantly increased glucose uptake and glycolytic capacity, suggesting that osteoblasts play an important role in glucose metabolism (Feigh et al., 2014). Further research found that GLP-1 significantly reduced blood glucose in osteoporosis rats accompanied by an increasing in osteocalcin synthesis, indicating that osteoporosis and diabetes have a potential pathophysiology mechanism, and diabetes treatment drugs may have effects on osteoporosis (Xu et al., 2017). Insulin and osteocalcin can promote secretion each other and have a positive correlation with each other. Large-scale clinical studies have also found that patients with osteoporosis often have elevated blood glucose levels, and even severe cases develop type 2 diabetes (Mulholland et al., 2005; Zeng et al., 2013). This study found that the blood glucose content in model rats was increased involved in starch, sucrose, and galactose metabolism, which may be caused by osteoporosis in the body and is consistent with the results of previous clinical studies. Imbalance of calcium metabolism is one of the common causes of osteoporosis. Especially in the elderly, the decrease in sex hormones and the relative increase in adrenal corticosterone not only affects bone synthesis but also has a certain effect on the absorption of calcium in the intestine leading to the increased excretion of calcium in feces, and the decreased calcium absorption capacity of renal tubule. With the gradual increase of age, the activity D gradually decreases, the parathyroid gland gradually increases, and the sex hormones gradually decrease. These changes cause increased osteoclast activity and decreased osteoblast activity (Delgado et al., 2016; Hirschberg et al., 2020). In postmenopausal women, in addition to the above factors, there are also the decline in estrone, estradiol, and estriol, which accelerate the decline in osteoblast activity, reduce bone matrix formation, and increase bone resorption (Fallah et al., 2013; Knuplez et al., 2020). Lysophosphatidylcholines (Lyso PC), also known as lysolecithin, is a class of phosphatidylcholine (PC) -derived compounds that are partially hydrolyzed by the removal of a FA group from PC. Lyso PC exists in the form of smaller phospholipids. Because of Lyso PC quickly metabolized by lysophospholipase and Lyso PC-acyltransferase, it exists for a very short time in the body (Vieira et al., 2019). Oxidized low density lipoprotein (Ox LDL) particles change bone cell function, and the main phospholipid component Lyso PC produced during LDL oxidation is cytotoxic to bone formation MG 63 osteoblast-like cells. It was found that Lyso PC can reduce the MG 63 osteoblast-like cell activity in a concentration-dependent manner. Lyso PC can also induce MG 63 osteoblast-like cell necrosis and apoptosis, and its cytotoxic effect is related to intracellular calcium homeostasis destruction (Brys et al., 2019). Corticosterone, estriol, and LysoPC (15: 0) of model animals were all down-regulated in this study indicating the occurrence of osteoporosis.

Functional metabolomics as a promising method could be used for the discovery of biomarkers to reveal the metabolic effect and potential targets of natural products (Wang et al., 2016; Qiu et al., 2017; Sun et al., 2018; Zhang et al., 2018; Wang et al., 2019; Zhang et al., 2019; Li et al., 2020; Qiu et al., 2020). The citric acid cycle is a process that release energy by oxidizing acetyl-Co A (Acetyl coenzyme A, acetyl-Co A) in all aerobic organisms. It provides certain amino acid precursors and the reducing agent NAD, which participates in many other biochemical reactions. NAD exists in the oxidized NAD+ forms and reduced NADH forms. TCA is widely found in the catabolism of proteins and fats. In protein catabolism, it is broken down into amino acids by proteases (Asnani et al., 2019). The citric acid content in the model group rats was decreased, suggesting that the TCA cycle and the metabolism of glyoxylic acid and dicarboxylic acid were abnormal in osteoporotic rats. Taurocholic acid is a conjugated bile acid, which can reduces inflammation, capillary permeability of inflammatory tissues, and inhibits the production of inflammatory mediators, such as NO and histamine. Taurocholic acid belongs to bile acid metabolism. Compared with the sham operation group, the taurocholic acid content is notably down-regulated with a significant difference. After ST administration, the taurocholic acid content is increased indicating that ST may improve osteoporosis by regulating anti-inflammatory effects. As the ultimate outcome of purine metabolism, uric acid is the primary index of purine metabolism. Blood uric acid is a lowering material in the human body, it has a strong antioxidant capacity in the redox reaction in the human body, which could scavenge free radicals to resist DNA damage (Wang et al., 2013). It has reported the physiological concentrations of uric acid in the blood possess anti-osteoporotic effects (Gormally et al., 2019; Lin et al., 2019; Kaushal et al., 2019). On the one hand, uric acid possess a certain protective effect on bone metabolism and is thought to be related to its antioxidant function. Uric acid is also associated with parathyroid hormone (PTH) levels in positive manner, and can also influence bone metabolism by adjusting the 1α-hydroxylase activity. On the other hand, oxidative stress can bring about bone loss and take part in the occurrence and development of osteoporosis, which may be the crystalline of uric acid is deposited. It reduces the activity of α-hydroxylase in the kidney and kidneys, and lower intestinal absorption of calcium (Chen et al., 2014).

## Conclusion

In summary, this research proved that there were obvious discrepancy between normal and osteoporosis rats, and evaluate efficacy of natural product osthole against osteoporosis rats by high throughput metabolomics analysis to discover biomarkers, metabolic profiles, and pathways as potential targets. Twenty-eight metabolites were highlighted as potential biomarkers of ovariectomized-osteoporosis. ST could cure osteoporosis by regulating 19 of them that were determined through metabolomics analysis. Combined with biochemical test, it found that ST effectively inhibits bone resorption and accelerates bone formation to enhance the therapeutic effect of osteoporosis in rats by affecting unsaturated fatty acid metabolism, lipid metabolism, amino acid metabolism, carbohydrate metabolism, TCA cycle, bile acid metabolism, as well as purine metabolism.

## Data Availability Statement

The raw data supporting the conclusions of this article will be made available by the authors, without undue reservation, to any qualified researcher.

## Ethics Statement

The animal study was reviewed and approved by The Animal Ethics Committee of Harbin Medical University ratify the experimental protocol and animals feeding method in this experiment.

## Author Contributions

SZ conceived and designed the experiments. ZSi, SZ, ZSh, and FL performed the experiment and analyzed the data. SZ guided the experiment. ZSi wrote the paper. All authors read and approved the final manuscript.

## Conflict of Interest

The authors declare that the research was conducted in the absence of any commercial or financial relationships that could be construed as a potential conflict of interest.
