# Supplementary material for: High-Throughput Metabolomics Discovers Metabolic Biomarkers and Pathways to Evaluating the Efficacy and Exploring Potential Mechanisms of Osthole Against Osteoporosis Based on UPLC/Q-TOF-MS Coupled With Multivariate Data Analysis
Source: Front Pharmacol. 2020 May 20;11:741. doi: 10.3389/fphar.2020.00741 (PMC7326133; doi:10.3389/fphar.2020.00741)
Supplement: Supplementary file 1 [file DataSheet_1.docx]

**Table S1** Significant biomarkers of differentially expressed metabolites in OP rats after ST treatment by metabolomics.

| **No.** | **Rt(min)** | **Mode** | **Name** | **HMDB code** | **Chemical formula** | **m/z** | **OVX** | **OVX+ALE** | **OVX+ST** | **Error** |
| --- | --- | --- | --- | --- | --- | --- | --- | --- | --- | --- |
| 1 | 0.58 | M-H | Lysine | HMDB0000182 | C6H14N2O2 | 145.10 | ↑ | ↓ | ⊕ | 1.6928 |
| 2 | 0.72 | M+H | Linoleic acid | HMDB0000673 | C18H32O2 | 281.25 | ↑ | ↓ | ⊕ | -0.2722 |
| 3 | 1.14 | M-H | 3-Hydroxybutyric acid | HMDB00357 | C4H8O3 | 103.04 | ↓ | ↑ | ⊕ | 2.7544 |
| 4 | 1.36 | M+H | Hippuric acid | HMDB00714 | C9H9NO3 | 180.06 | ↑ | ↓ |  | 1.3502 |
| 5 | 1.69 | M-H | 9E,11E-Octadecadienoic acid | HMDB0005047 | C18H32O2 | 279.23 | ↑ | ↓ |  | -1.3125 |
| 6 | 1.92 | M-H | Prostaglandin F2a | HMDB0001139 | C20H34O5 | 399.24 | ↑ | ↓ | ⊕ | 1.4901 |
| 7 | 2.33 | M-H | Taurocholic acid | HMDB00036 | C26H45NO7S | 514.28 | ↓ | ↑ | ⊕ | 3.2343 |
| 8 | 2.61 | M+H | LysoPC(15:0) | HMDB10381 | C23H48NO7P | 482.32 | ↓ | ↑ | ⊕ | 1.9888 |
| 9 | 2.84 | M+H | L-Carnitine | HMDB0000062 | C_7_H_15_NO_3_ | 162.1141 | ↑ | ↓ | ⊕ | 1.3378 |
| 10 | 2.99 | M+H | Glucose | HMDB00122 | C6H12O6 | 181.0714 | ↑ | ↓ | ⊕ | -2.9741 |
| 11 | 3.39 | M+H | Arginine | HMDB03416 | C6H14N4O2 | 175.1193 | ↑ | ↓ | ⊕ | 2.2435 |
| 12 | 3.62 | M-H | Citric acid | HMDB00094 | C6H8O7 | 191.0198 | ↓ | ↑ | ⊕ | -1.3542 |
| 13 | 3.90 | M-H | Corticosterone | HMDB01547 | C21H30O4 | 345.2052 | ↓ | ↑ | ⊕ | -3.5676 |
| 14 | 4.28 | M+H | S-Adenosylhomocysteine | HMDB00939 | C14H20N6O5S | 407.11 | ↑ | ↓ |  | 3.8609 |
| 15 | 4.56 | M-H | Ornithine | HMDB00214 | C5H12N2O2 | 131.08 | ↑ | ↓ | ⊕ | 3.2132 |
| 16 | 4.75 | M+H | Tryptophan | HMDB13609 | C11H12N2O2 | 205.10 | ↑ | ↓ | ⊕ | 1.1123 |
| 17 | 4.90 | M+H | Arachidonic acid | HMDB0001043 | C20H32O2 | 305.25 | ↑ | ↓ | ⊕ | -7.5142 |
| 18 | 5.22 | M+H | 18-Hydroxyarachidonic acid | HMDB0006245 | C20H32O3 | 321.24 | ↑ | ↓ |  | -4.7807 |
| 19 | 5.30 | M+H | Methionyl-Hydroxyproline | HMDB28974 | C10H18N2O4S | 263.11 | ↑ | ↓ |  | 1.6928 |
| 20 | 6.00 | M-H | Cer(d18:0/18:0) | HMDB11761 | C36H73NO3 | 566.55 | ↓ | ↑ | ⊕ | -4.7520 |
| 21 | 6.41 | M-H | Docosahexaenoic acid | HMDB0002183 | C22H32O2 | 327.23 | ↓ | ↑ |  | -8.2258 |
| 22 | 6.51 | M-H | Palmitic acid | HMDB0000220 | C16H32O2 | 255.23 | ↓ | ↑ |  | -5.2631 |
| 23 | 6.71 | M+H | 4-Oxoretinol | HMDB0012329 | C20H28O2 | 301.22 | ↓ | ↑ |  | 1.1284 |
| 24 | 7.09 | M-H | Glyceraldehyde | HMDB0001051 | C3H6O3 | 89.02 | ↓ | ↑ |  | 3.0329 |
| 25 | 7.73 | M-H | Glutamine | HMDB00641 | C5H10N2O3 | 145.06 | ↓ | ↑ | ⊕ | 2.3434 |
| 26 | 8.08 | M+H | Uric acid | HMDB00289 | C5H4N4O3 | 169.04 | ↓ | ↑ | ⊕ | 5.7271 |
| 27 | 8.31 | M+H | 8-HETE | HMDB04679 | C20H32O3 | 321.24 | ↓ | ↑ | ⊕ | 6.9467 |
| 28 | 9.69 | M-H | Estriol | HMDB0000153 | C_18_H_24_O_3_ | 311.1618 | ↑ | ↓ | ⊕ | 3.4462 |


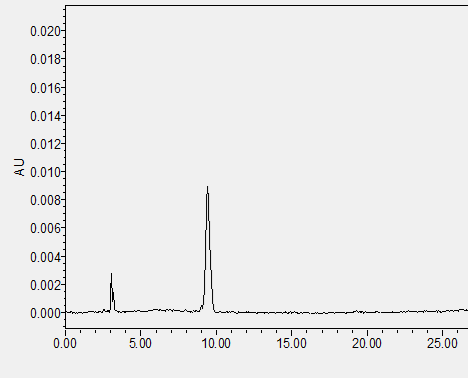


Fig. S1 HPLC characteristic chromatography of Osthole. HPLC was performed on the column of Kromasil C18 column. The mobile phase was water -acetonitrile system (30:70) at the flow rate of 1.0 ml/min. The column temperature was 25 ^o^C, and the injection volume was 10 ul. The detection wavelength were set at 322 nm.
